# Radiomics and Machine Learning Analysis Based on Magnetic Resonance Imaging in the Assessment of Colorectal Liver Metastases Growth Pattern

**DOI:** 10.3390/diagnostics12051115

**Published:** 2022-04-29

**Authors:** Vincenza Granata, Roberta Fusco, Federica De Muzio, Carmen Cutolo, Mauro Mattace Raso, Michela Gabelloni, Antonio Avallone, Alessandro Ottaiano, Fabiana Tatangelo, Maria Chiara Brunese, Vittorio Miele, Francesco Izzo, Antonella Petrillo

**Affiliations:** 1 Division of Radiology, Istituto Nazionale Tumori IRCCS Fondazione Pascale—IRCCS di Napoli, 80131 Naples, Italy; m.mattaceraso@istitutotumori.na.it (M.M.R.); a.petrillo@istitutotumori.na.it (A.P.); 2 Medical Oncology Division, Igea SpA, 41012 Carpi, Italy; r.fusco@igeamedical.com; 3 Diagnostic Imaging Section, Department of Medical and Surgical Sciences & Neurosciences, University of Molise, 86100 Campobasso, Italy; demuziofederica@gmail.com (F.D.M.); m.brunese@studenti.unimol.it (M.C.B.); 4 Department of Medicine, Surgery and Dentistry, University of Salerno, 84084 Fisciano, Italy; carmencutolo@hotmail.it; 5 Diagnostic and Interventional Radiology, University Hospital of Pisa, Via Paradisa 2, 56100 Pisa, Italy; michela.gabelloni@gmail.com; 6 Division of Abdominal Oncology, Istituto Nazionale Tumori IRCCS Fondazione Pascale—IRCCS di Napoli, 80131 Naples, Italy; a.avallone@istitutotumori.na.it (A.A.); a.ottaiano@istitutotumori.na.it (A.O.); 7 Division of Pathology, Istituto Nazionale Tumori IRCCS Fondazione Pascale—IRCCS di Napoli, 80131 Naples, Italy; f.tatangelo@istitutotumori.na.it; 8 Division of Radiology, Azienda Ospedaliera Universitaria Careggi, 50134 Firenze, Italy; vmiele@sirm.org; 9 Italian Society of Medical and Interventional Radiology (SIRM), SIRM Foundation, 20122 Milan, Italy; 10 Division of Epatobiliary Surgical Oncology, Istituto Nazionale Tumori IRCCS Fondazione Pascale—IRCCS di Napoli, 80131 Naples, Italy; f.izzo@istitutotumori.na.it

**Keywords:** EOB-MRI study, colorectal liver metastases, radiomics

## Abstract

To assess Radiomics and Machine Learning Analysis in Liver Colon and Rectal Cancer Metastases (CRLM) Growth Pattern, we evaluated, retrospectively, a training set of 51 patients with 121 liver metastases and an external validation set of 30 patients with a single lesion. All patients were subjected to MRI studies in pre-surgical setting. For each segmented volume of interest (VOI), 851 radiomics features were extracted using PyRadiomics package. Nonparametric test, univariate, linear regression analysis and patter recognition approaches were performed. The best results to discriminate expansive versus infiltrative front of tumor growth with the highest accuracy and AUC at univariate analysis were obtained by the wavelet_LHH_glrlm_ShortRunLowGray Level Emphasis from portal phase of contrast study. With regard to linear regression model, this increased the performance obtained respect to the univariate analysis for each sequence except that for EOB-phase sequence. The best results were obtained by a linear regression model of 15 significant features extracted by the T2-W SPACE sequence. Furthermore, using pattern recognition approaches, the diagnostic performance to discriminate the expansive versus infiltrative front of tumor growth increased again and the best classifier was a weighted KNN trained with the 9 significant metrics extracted from the portal phase of contrast study, with an accuracy of 92% on training set and of 91% on validation set. In the present study, we have demonstrated as Radiomics and Machine Learning Analysis, based on EOB-MRI study, allow to identify several biomarkers that permit to recognise the different Growth Patterns in CRLM.

## 1. Introduction

Colorectal cancer (CRC) is one of the main common cancer worldwide, representing about the 10% of new detected cancers in 2020 [1]. Moreover, it is estimated that in 2040 its prevalence will rise quickly to >3 million cases per year [2,3]. Although the management of patients within a multidisciplinary team have improved the clinical outcome thanks to a closer patient follow-up to obtain an earlier detection of metastatic disease and an improvement in the efficacy of systemic therapies, and of surgical indications and –quality, based on a better patient selection, however metastatic spread is the main cause of death [4,5,6,7,8]. In addition, at the diagnosis, about 20% of patients have liver metastases (CRLM), while approximately 40–50% of patients will develop CRLM during surveillance, either after primary tumor resection/multimodal therapy or after the resection of CRLM [8,9,10,11,12,13,14,15,16]. Another key is related to the fact that about 60% of patients can expect to have liver recurrence even after a R0 resection of the primary CRLM. Several researches have demonstrated as patients with (a) T3/T4 CRC status, (b) local positive node, and more than 3 CRLM have higher possibility of recurrence [17,18,19]. In addition, several researches have suggested that the distinct growth pattern of CRLM could be linked to liver recurrence and overall survival (OS) after conversion therapy and resection [20,21,22]. With the increasing frequency of CRLM resections, the histology of the front morphology of CRLM is accessible and different growth patterns have been reported: in the “desmoplastic” or “encapsulated” model, tumour cells are separated from the liver parenchyma by a fibrotic stroma border that is distinguished by a ‘pushing’ or ‘expansive’ pattern, in which the hepatic plaques adjacent to the metastases are flattened with no intermediate fibrotic tissue. The model called “invasive” or “replacement” in which cancer cells infiltrate normal surrounding liver parenchyma, replace the hepatocytes and the sinusoidal stromal [20,21,22]. A recent review [22] assessed 17 clinical studies, including either chemonaïve and chemotherapy-treated patients, showed that in the 82.4% a statistically significant favourable outcome was reported for patients with desmoplastic CRLM while in the 66.7% a significantly unfavourable outcome for patients with a predominantly replacement-type CRLM was demonstrated [22].

In this context, it is evident that the possibility to identify biomarkers that allow, during imaging studies, a proper growth pattern characterization consents a better patient treatment selection [23,24,25,26,27,28,29]. Since, radiomics allows to evaluate tissue at microscopic level, in order to obtain quantitative data to employ as biomarkers [30,31,32,33,34,35,36,37,38,39], this analysis allow to increase diagnostic, prognostic and predictive accuracy in oncological setting [40,41,42,43,44,45,46,47,48]. In fact, Radiomic is created to be employed in decision support of precision medicine, using standard of care images [49,50,51,52,53,54,55,56,57]. Although, several researches have assessed the MRI features of CRLM [58,59,60], at the best of our knowledge no study have evaluated the ability of Radiomics analysis based on MRI studies to characterize CRLM growth pattern. The purpose of this study is to assess the Radiomics and Machine Learning Analysis Based on MRI in the evaluation of CRLM growth pattern.

## 2. Materials and Methods

Local Ethical Committee board accepted this retrospective study, renouncing to the patient informed consent. Patients were selected by radiological database considering the temporal period from January 2018 to June 2021 and the following inclusion criteria: (1) liver pathological proven metastases; (2) MRI study of high quality in pre-surgical setting and (3) a follow-up Computed Tomography (CT) scan of at least six months after surgical liver resection.

The patient cohort included a training set of 51 patients with 121 liver metastases, and an external validation set of 30 patients with a single lesion. The validation cohort was provided by “Careggi Hospital”, Florence, Italy. The patient characteristics were reported in Table 1.

### 2.1. MR Imaging Protocol

MR studies were performed with two 1.5T MR scanners: Magnetom Symphony (Siemens, Erlangen, Germany) and Magnetom Aera (Siemens).

The MRI study protocol included conventional sequences, T1 weighted (W), without contrast medium administration, and T2-W, Diffusion Weighted Imaging (DWI) with seven b values in order to obtain functional parameters with mono-exponential model and T1-W sequences after the administration of contrast medium. In Table 2 we reported MR study protocol.

According to the different phase of patient management, our study protocol includes the possibility to administrate a liver-specific contrast agent (in pre surgical setting) and a non-liver-specific contrast agent (in characterization and staging phase). In this study we assessed images obtained employing a liver-specific contrast agent (0.1 mL/kg of Gd-EOB-BPTA—Primovist, Bayer Schering Pharma, Berlin, Germany). A power injector (Spectris Solaris^®^ EP MR, MEDRAD Inc., Indianola, IA, USA) was used to administrate the contrast agent using an infusion rate of 2 mL/s.

After contrast medium administration, VIBE T1-weighted FS (SPAIR) sequences were acquired in different phases of contrast study: arterial (35 s delay), portal/venous (90 s), transitional (120 s), and hepatospecific (EOB) phase (20 min).

### 2.2. MRI Post-Processing

For each volume of interest, 851 radiomic features were extracted as median values on the segmented volume by two expert radiologists in abdominal imaging and MRI, using the PyRadiomics tool [61] and as previously reported in [62,63] and as reported in [https://readthedocs.org/projects/pyradiomics/downloads/ (accessed on 21 December 2021)].

### 2.3. Reference Standard

Histopathologic data, from routine report were used as the reference standard for determining metastases growth pattern. Lesions with desmoplastic growth pattern were defined as expansive; lesions with predominantly replacement-type were defined infiltrative.

### 2.4. Statistical Analysis

Intraclass correlation coefficient was used to evaluate the variability by two radiologists. The non-parametric Kruskal-Wallis test was performed to recognize differences statistically significant of radiomics metrics median values in the identification of tumor growth front (expansive versus infiltrative).

Chi square test was used to evaluate if the tumor growth front (expansive versus infiltrative) was correlated with the local recurrence occurrence.

Receiver operating characteristic (ROC) and Youden index was used to calculate the optimal cut-off for each metric and area under curve (AUC), sensitivity, specificity, positive predictive value (PPV), negative predictive value (NPV) and accuracy.

A feature selection was made to delete the redundant and non informative metrics considering the following findings: metrics significant by Kruskal-Wallis test and with an accuracy when considered alone major of 75%. A linear regression analysis was made to calculate the linear regression model of all significant metrics.

Moreover, approaches of artificial intelligence were considered to define the best classifier of all significant metrics. The classifiers considered included support vector machine (SVM), k-nearest neighbors (KNN), artificial neural network (NNET), and decision tree (DT)) [63]. The best model was selected considering the highest area under ROC curve and the highest accuracy. Each classifier was trained with a 10-k fold cross validation and was validated using the external validation set.

McNemar test was used to evaluate that the results of the dichotomy tables were statistically significant.

A *p*-value < 0.05 was considered as significant. The analysis was made considering the Statistics and Machine Toolbox of MATLAB R2021b (MathWorks, Natick, MA, USA).

## 3. Results

No correlation statistically significant was relived between the tumor growth front and the local recurrence occurrence (*p*-value = 0.82 at Chi square test).

On univariate analysis (Table 3), a variable number of metrics were statistically significant which were different when extracted from different MR sequences: 15 significant predictors extracted from T2W SPACE; 8 significant predictors extracted from the arterial phase; 9 significant predictors extracted from the portal phase; 8 significant predictors extracted from the EOB phase.

The best results (Table 3) with the highest accuracy and AUC at univariate analysis to discriminate expansive versus infiltrative front of tumor growth were obtained by the wavelet_LHH_glrlm_ShortRunLowGrayLevelEmphasis from portal phase sequence with accuracy of 82%, sensitivity of 84%, specificity of 77%, PPV and NPV of 85% and 74%, respectively, and a cut-off value of 0.12. The results were statistically different from those obtained with the T2W and arterial phase MR sequences while were to those obtained from EOB phase sequence with the wavelet_HLL_glcm_InverseVariance that obtained and AUC of 78% and an accuracy of 83%.

Linear regression model increased the performance obtained respect to the univariate analysis (see Table 4) for each sequence except that for EOB-phase sequence. The best results were obtained by a linear regression model of 15 significant features extracted by the T2W SPACE sequence with accuracy of 90%, a sensitivity of 95%, a specificity of 95%, a PPV and a NPV of 80% and 89% respectively. These results were statistically different from the univariate analysis results and compared with the results of metrics extracted from other MR sequences (*p*-value < 0.01 on McNemar’s test). Table 5 reported the coefficients of metrics and intercept of the best linear regression model.

Furthermore, using pattern recognition approaches, the diagnostic performance to discriminate the expansive versus infiltrative front of tumor growth increased again and the best classifier was a weighted KNN trained with the 9 significant metrics extracted from the portal phase sequence obtaining 92% accuracy on training set and 91% at validation set with an AUC of 0.97 and 0.99, respectively analysis (see Table 6). These results were statistically different from the univariate analysis results and linear regression analysis (*p*-value < 0.01 on McNemar’s test).

**Figure 1:**
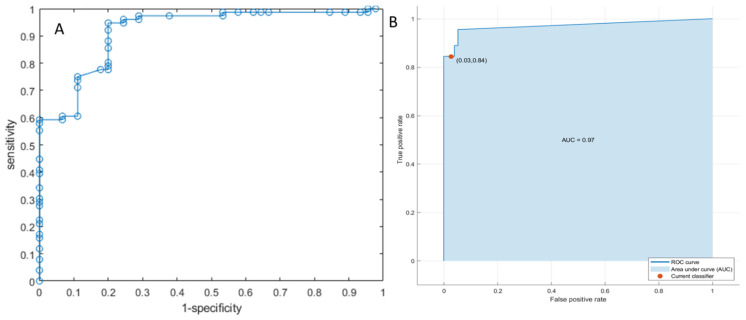
ROC curve of linear regression model of 15 significant features by T2w sequence in (**A**) and ROC curve of a KNN trained with the 9 significant predictors extracted from the portal phase in (**B**).

Figure 1 showed the ROC curve of linear regression model of 15 significant features by T2w sequence and the ROC curve of a KNN trained with the 9 significant predictors extracted from the portal phase.

All results of the dichotomy tables were statistically significant (*p*-value < 0.01 at McNemar test).

## 4. Discussion

Since liver is the most common site of distant metastases in patients with CRC and, surgical resection is the only curative treatment, it is clear that the possibility to identify several prognostic features of CRLM to guide the proper patient management, remains a critical key open question. In addition, according to current principles for oncologic liver surgery, liver resection aims to remove the lesion with adequate margins and with sufficient liver remnant volume. This needs a multi-parametric patient assessment for a correct evaluation of the lesion and functional liver status [56,57,58]. Several features could guide the surgical procedure, comprising liver functional status, patient general status, tumour size, localization, and vascular infiltration.

Also, today, several researches have suggested that the distinct CRLM growth pattern is associated with differences in tumor local recurrence and OS. The rationale behind the need to know CRLM growth patterns is at least twofold: on one hand, the patterns may be useful for prognostication and may aid treatment decisions; on the other hand, it is likely that understanding these distinct patterns of metastatic progression will provide important insights into the biological mechanisms that support tumour growth in the liver [22]. Fernández Moro et al. [22] reported that microvessel co-option, besides providing vascular supply, renders metastases of the replacement type resistant to anti-angiogenic therapy. Furthermore, the authors suggested an alternative approach for treating replacement-type metastases. It is worth noting that several studies published before FDA approval of anti-angiogenic therapy found a favourable prognosis for patients with desmoplastic-type CRLM, suggesting that there are important mechanistic insights beyond differences in angiogenesis to be gained from the different growth patterns [22].

In this scenario, it is clear that having the possibility, during an imaging study, to identify biomarkers that could correlate with the growth of the lesion, allows for better treatment selection [58,59,60]. At the best of our knowledge no one study has evaluated the Radiomics and Machine Learning Analysis Based on MRI study, in the assessment of CRLM growth pattern. With regard to univariate analysis, a variable number of metrics were statistically significant: 15 extracted from T2W SPACE; 8 from the arterial phase; 9 extracted from the portal phase; 8 extracted from the EOB phase. The best results with the highest accuracy and AUC at univariate analysis to discriminate expansive versus infiltrative front of tumor growth were obtained by the wavelet_LHH_glrlm_ShortRunLowGrayLevelEmphasis from portal phase.

With regard to linear regression model, this increased the performance obtained respect to the univariate analysis for each sequence except that for EOB-phase sequence. The best results were obtained by a linear regression model of 15 significant features extracted by the T2W SPACE. These results were statistically different from the univariate analysis results. Furthermore, using pattern recognition approaches, the diagnostic performance to discriminate the expansive versus infiltrative front of tumor growth increased again and the best classifier was a weighted KNN trained with the 9 significant metrics extracted from the portal phase. Our results were confirmed by validation external dataset.

However, when we evaluated the tumor growth pattern and the local recurrence, we found no statistically significant correlation. This result could be explained by the number of patients under examination and the short follow-up period.

To date, several researches have assessed Radiomics analysis and CRLM, with regard to mutational status, prognosis and recurrence [32,33,64,65,66,67,68,69,70,71,72,73,74,75,76,77,78,79,80,81]. Andersen et al. showed as homogeneity features were correlated to worse OS [65]. Lubner et al. assessed KRAS status, showing an inversely correlation with the skewness degree, while they showed an association between entropy and OS [67]. In our previous studies we showed that radiomics features obtained by EOB-MRI phase, arterial and portal phase so as by T2-W sequences, allow to predict clinical outcomes following liver resection in CRLM [80,81].

This study has several weaknesses: (1) the small sample size, although the analysis was done on a homogeneous group and on all single lesion; (2) the retrospective nature, (3) a manual segmentation. Furthermore, we not evaluated: (4) the impact of chemotherapy on our data, while we assessed all single study protocol phase, in order to identify the more accurate sequence in the assessment of growth pattern and our results were confirmed by a validation external dataset.

## 5. Conclusions

In the present study, we have demonstrated as Radiomics and Machine Learning Analysis, based on EOB-MRI study, allow to identify several biomarkers that permited to recognise the different Growth Patterns in CRLM. 

The best results with the highest accuracy and AUC at univariate analysis were obtained by the wavelet_LHH_glrlm_ShortRunLowGrayLevelEmphasis from portal phase. Linear regression model increased the performance obtained respect to the univariate analysis for each sequence, except that for EOB-phase sequence. Furthermore, using pattern recognition approaches, the best classifier was a weighted KNN trained with the 9 significant metrics extracted from the portal phase.

## Figures and Tables

**Table 1:** Characteristics of the study population (81 patients).

Patient Description	Numbers (%)/Range
Gender	Men 53 (65.4%)
	Women 28 (34.6%)
Age	61 y; range: 35–82 y
Primary cancer site	
Colon	52 (64.2%)
Rectum	29 (35.8%)
Prior Chemotherapy	81 (100%)
Hepatic metastases description	
Patients with single nodule	52 (64.2%)
Patients with multiple nodules	29 (35.8%)/range: 2–13 metastases
Nodule size (mm)	mean size 36.4 mm; range 7–58 mm
Mucinous carcinoma	25 (30.9%)
RAS mutation	42 (51.9%)
Liver Recurrence	19 (23.5%)

**Table 2:** MR acquisition protocol.

Sequence	Orientation	TR/TE/FA (ms/ms/deg.)	AT (min)	Acquisition Matrix	ST/Gap (mm)	FS
Trufisp T2-W	Coronal	4.30/2.15/80	0.46	512 × 512	4/0	without
HASTE T2-W	Axial	1500/90/170	0.36	320 × 320	5/0	Without and with (SPAIR)
HASTE T2w	Coronal	1500/92/170	0.38	320 × 320	5/0	without
SPACE T2W FS	Axial	4471/259/120	4.20	384 × 450	3/0	With (Spair)
In-Out phase T1-W	Axial	160/2.35/70	0.33	256 × 192	5/0	without
DWI	Axial	7500/91/90	7	192 × 192	3/0	without
Vibe T1-W	Axial	4.80/1.76/30	0.18	320 × 260	3/0	with (SPAIR)

Note: TR = Repetition time, TE = Echo time, FA = Flip angle, AT = Acquisition time, ST = Slice thickness, FS = Fat suppression, SPAIR = Spectral adiabatic inversion recovery.

**Table 3:** Univariate analysis results to predict mucinous type.

	T2W SPACE	Arterial Phase	Portal Phase	EOB-Phase
	wavelet_HLL_firstorder_Median	wavelet_LHH_glrlm_ShortRunLowGrayLevelEmphasis	wavelet_LHH_glrlm_ShortRunLowGrayLevelEmphasis	wavelet_HLL_glcm_InverseVariance
AUC	0.71	0.69	0.80	0.78
Sensitivity	0.79	0.95	0.84	0.84
Specificity	0.73	0.51	0.77	0.82
PPV	0.83	0.77	0.85	0.89
NPV	0.67	0.85	0.74	0.76
Accuracy	0.77	0.79	0.82	0.83
Cut-off	−0.39	0.12	0.12	0.46

**Table 4:** Linear regression with significant features.

Linear Regression of Significant Features Extracted by	AUC	Sensitivity	Specificity	PPV	NPV	Accuracy	Cut-Off
T2W SPACE	0.90	0.95	0.80	0.89	0.90	0.89	1.51
arterial phase	0.74	0.89	0.89	0.93	0.83	0.89	1.45
portal phase	0.88	0.80	0.89	0.92	0.73	0.83	1.58
EOB-phase	0.55	0.88	0.56	0.77	0.74	0.76	8.81

**Table 5:** Linear regression model coefficients.

Features	Coefficients	*p*-Value
Intercept	−10.99	0.01
original_shape_SurfaceVolumeRatio	−1.13	0.24
wavelet_HLL_glcm_InverseVariance	13.96	0.01
wavelet_HLL_firstorder_Median	0.14	0.06
wavelet_HLL_glrlm_ShortRunEmphasis	38.72	0.00
wavelet_HLL_glrlm_RunPercentage	−38.39	0.00
wavelet_LHL_gldm_DependenceNonUniformityNormalized	−7.33	0.61
wavelet_LHL_glcm_InverseVariance	−3.19	0.51
wavelet_LHL_firstorder_Kurtosis	0.01	0.04
wavelet_LHL_glrlm_ShortRunEmphasis	−24.29	0.21
wavelet_LHL_glrlm_RunPercentage	46.40	0.00
wavelet_LHL_glrlm_RunLengthNonUniformityNormalized	−14.58	0.15
wavelet_LLH_glcm_Imc1	−0.31	0.87
wavelet_LLL_firstorder_Uniformity	6.76	0.17
wavelet_LLL_firstorder_Minimum	0.01	0.00
wavelet_LLL_glrlm_GrayLevelNonUniformityNormalized	−5.61	0.28

**Table 6:** Pattern recognition analysis with significant features.

The Best Classifier (KNN) Results with Significant Features Extracted by	Dataset	AUC	Accuracy	Sensitivity	Specificity	Training Time [s]	Model Type and Parameters
T2W SPACE	Training set	0.90	0.89	0.84	0.92	11.1	Decision Fine Tree; Maximum number of splits: 100; split criterion: Gini’s diversity index; optimizer options: Hyperparameter options disabled
	Validation set	0.88	0.86	0.86	0.86		
arterial phase	Training set	0.97	0.91	0.91	0.91	2.34	Weighted KNN; number of neighbors: 10; distance metric: Euclidean; distance weight: squared inverse
	Validation set	0.96	0.89	0.85	0.91		
portal phase	Training set	0.97	0.92	0.84	0.97	9.74	
	Validation set	0.99	0.91	0.81	0.96		
EOB-phase	Training set	0.96	0.90	0.91	0.89	13.4	
	Validation set	0.95	0.80	0.67	1.00		

## Data Availability

Some data are available at link https://zenodo.org/record/6496965#.Ympr5tpBy3A.
